# RE: Water pipe (shisha) smoking among male students of medical colleges in the eastern region of Saudi Arabia

**DOI:** 10.4103/0256-4947.75795

**Published:** 2011

**Authors:** Abdullah M. Al-Bedah, Naseem A. Qureshi

**Affiliations:** From the a Arabian Center for Tobacco Control, and Ministryof Health, Riyadh, Saudi Arabia; b General Administration for Medical Research & Mental Health and Social Services, Ministry of Health, Riyadh, Saudi Arabia

**To the Editor:** We have critically read the brief report by Dr. Taha et al^1^ and offer some comments that might be useful to the readers of this journal. Evidently, shisha smoking is a major public health problem and this socializing but highly dangerous toxic behavior has spread worldwide to include the African and Asian continents, Australia, Europe, and North America. Therefore, water pipe smoking is no more confined to the Arabic and or Eastern world. Secondly, use of the water pipe is potentially hazardous and by all yardsticks more dangerous than cigarette smoking. In addition, second hand smoke from the water pipe is thought to be more dangerous than that from cigarette smoke, although opposing evidence has also been reported.^2^ Shisha smoking is reported to contribute to a variety of diseases including cardiovascular, respiratory, addiction, and cancer. The lingering misbelief among the general public that water pipe smoking is less dangerous than cigarette smoking is no longer tenable.^3^ Thirdly, a critique of relevant literature on tobacco use goes hand in hand with the saying that everything in excess is very bad. An epidemiological study is needed to assess mild to moderate use of water pipe and tobacco use in the general population. There is currently converging evidence that water pipe smoking is generally more common among young adolescents, including girls, at a global level. This is a very dangerous epidemiological trend in the young, who already have other compounding lifestyle problems that might result in disastrous consequences.

Unfortunately, this study recruited only male medical students and hence lost an opportunity to explore simultaneously the water pipe smoking behavior of female medical students. This is a major caveat because a comparative study involving both genders might have substantiated or refuted already emerging findings; one of them is that water pipe smoking is more common among females. Another limitation of this study is that the authors designed a new questionnaire rather than using one of the most relevant standardized questionnaires developed jointly by international health organizations, including the World Health Organization, the Canadian Public Health Association and the US Centers for Disease Control and Prevention. In doing so, the authors fell short of assessing the specific and relative social and cultural beliefs of male versus female students in Saudi Arabia, as medical students in medical colleges often hail from different regions. Furthermore, this study did not add anything major or new to the existing literature on shisha smoking. However, one finding and related explanation is noteworthy: mothers of water pipe smokers had an advanced education compared with mothers of those of nonsmokers. According to this study this result was speculatively attributed to two factors including shisha smoking as a prestige behavior and an indication of a modern standard of living. However, the commentators feel that these explanations might not be true because mothers were not asked about the explanations underlying the linkage between their higher education and shisha smoking. This finding needs further study. There might be more plausible explanations: shisha smoking in modern times is merely a continuation of a very old traditional behavior of the Eastern world and it may not be a prestigious behavior at all; rather it may reflect low status. A study that recruits a larger sample representative of all Saudi medical colleges might truly shed light on this finding and a more detailed explanation must be included in the measurement tools.^4^

Finally, shisha smoking is a global health problem of the young population and needs effective preventive strategies across the world, including health warning labels on water pipe tobacco products and related accessories.^5^ Shisha smoking is a rapidly re-emerging epidemic, especially among the young population and hence is a research priority. In addition, there are some grey areas, especially epidemiological perspectives and long-term effects of water pipe smoking, including cancer development, that need the special attention of researchers worldwide.

The author of the original report declined to respond
